# A Genetic Polymorphism in the *WDR72* Gene is Associated With Calcium Nephrolithiasis in the Chinese Han Population

**DOI:** 10.3389/fgene.2022.897051

**Published:** 2022-07-14

**Authors:** Lujia Wang, Zijian Zhou, Yuanyuan Yang, Peng Gao, Xiaoling Lin, Zhong Wu

**Affiliations:** ^1^ Department of Urology, Huashan Hospital & Institute of Urology, Fudan University, Shanghai, China; ^2^ Clinical Research Center of Urolithiasis, Shanghai Medical College, Fudan University, Shanghai, China; ^3^ Department of Urology, Shanghai Children’s Hospital, School of Medicine, Shanghai Jiao Tong University, Shanghai, China

**Keywords:** nephrolithiasis, *WDR72*, *DGKH*, *CLDN14*, *SLC34A1*, *HIBADH*, CaSR signaling, Chinese Han population

## Abstract

A previous genome-wide association study (GWAS) reported several novel loci for nephrolithiasis in British and Japanese population, some of which were predicted to influence CaSR signaling. In this study, we aimed to evaluate the association of these loci with calcium nephrolithiasis in Chinese Han population. We performed a case-control association analysis involving 691 patients with calcium nephrolithiasis and 1008 control subjects. We were able to genotype a total of 17 single-nucleotide polymorphisms (SNPs), which were previously reported to be significantly associated with nephrolithiasis in GWAS. rs578595 at *WDR*72 was significantly associated with calcium nephrolithiasis in Chinese Han population (*p* < 0.001, OR = 0.617). Moreover, rs12654812 at *SLC34A1* (*p* = 0.0427, OR = 1.170), rs12539707 at *HIBADH* (*p* = 0.0179, OR = 0.734), rs1037271 at *DGKH* (*p* = 0.0096, OR = 0.828) and rs12626330 at *CLDN14* (*p* = 0.0080, OR = 1.213) indicated suggestive associations with calcium nephrolithiasis. Our results elucidated the significance of genetic variation at *WDR*72, *DGKH*, *CLDN14*, *SLC34A1*, and *HIBADH* in Chinese patients with nephrolithiasis. Since polymorphisms of *WDR72*, *DGKH*, and *CLDN14* are predicted to influence in CaSR signaling, our results emphasized the role of abnormal calcium homeostasis in calcium nephrolithiasis.

## Introduction

Nephrolithiasis is one of the most frequent disorders affecting almost all populations. Epidemiological studies have reported that the incidence of nephrolithiasis is about 5% among females and 12% among males (Lewandowski and Rodgers, 2004), and almost 70% of all kidney stones are composed of calcium oxalate and/or phosphate (Croppi et al., 2012; Liu et al., 2021). In recent years, the incidence and prevalence of nephrolithiasis is increasing globally. Nephrolithiasis is commonly recurrent, with up to 50% of individuals experiencing a second episode within 10 years of the initial presentation, and recurrent stone disease has been linked to renal function decline (Pearle et al., 2014; Yan et al., 2021). The cause of nephrolithiasis is considered multifactorial, including but not limited to diet, ethnic, climate, and genetic factors. Recent studies estimated that up to 65% of kidney stone formers have a family history of nephrolithiasis (Stechman et al., 2007). Moreover, twin studies have reported a heritability of >45% for nephrolithiasis, and a strong family history of nephrolithiasis, including a parent and two siblings, have a standard incidence ratio for stone formation of >50 (Hemminki et al., 2018).

Up till now, six genome-wide association studies (GWAS) in different ethnicity have identified about 25 loci associated with nephrolithiasis (Thorleifsson et al., 2009; Gudbjartsson et al., 2010; Urabe et al., 2012; Oddsson et al., 2015; Howles et al., 2019; Tanikawa et al., 2019). Loci identified by GWAS in patients with nephrolithiasis could provide possible insight into the pathogenesis of the disorder. Howles et al. (2019) reported a trans-ethnic GWAS meta-analysis of British and Japanese cohorts, which identified 7 novel loci associated with nephrolithiasis. Among them, five of the loci, *WDR72*, *GPIC1*, *DGKD*, *DGKH*, and *BCR*, were predicted to influence calcium-sensing receptor (CaSR) signaling. The CaSR is a G protein-coupled receptor, which is highly expressed in the kidneys and parathyroid (Litvinova et al., 2021). CaSR has a central role in calcium hemostasis, including increasing kidney calcium reabsorption while stimulating parathyroid hormone (PTH) release to enhance bone resorption, urinary calcium reabsorption and 1,25-dihydroxyvitamin D_3_ synthesis in the kidney (Hannan et al., 2016). In this study, we conducted the research regarding the association between polymorphisms of those CaSR-related genes and calcium nephrolithiasis in Chinese Han population.

## Materials and Methods

### Subjects

In total, 691 unrelated Chinese Han patients with nephrolithiasis (467 males and 224 females, mean aged 50.47 years) were recruited at Huashan Hospital of Fudan University. Patients with nephrolithiasis secondary to known causes, such as chronic kidney disease, renal failure, chronic diarrhea, gout, renal tubular acidosis, primary and secondary hyperparathyroidism, osteoporosis, or cancer were excluded. Patients with radioparent stones, including struvite, uric acid and cystine stones were excluded. We also excluded patients with history of medications that affected urinary calcium excretion or taking vitamin D and/or calcium supplements. The control group consisted of 1008 subjects were age/gender matched individuals without a history of nephrolithiasis or a family history of kidney stone disease. Nephrolithiasis was diagnosed clinically either with plane radiography of kidney-ureter-bladder (KUB) or non-contrast computed tomography (CT) scan. All the patients with nephrolithiasis and the control subjects were of the same racial, ethnic, geographical and environmental strata.

We assessed the effect of genetic variations on serum calcium, sodium, potassium, magnesium, phosphorus, chloride, carbon dioxide (CO_2_), creatinine, urea, uric acid, alkaline phosphatase (ALP), parathyroid hormone (PTH), serum 25-hydroxycholecalciferol, albumin, glucose, cholesterol, triglycerides, low-density lipoprotein (LDL), high-density lipoprotein (HDL) levels; urine calcium and phosphorus levels; estimated glomerular filtration rate (eGFR); and body mass index (BMI). Laboratory measurements were performed within 2 weeks preoperatively.

### Blood Sample Collection and DNA Extraction

Peripheral blood samples were collected by venipuncture in a tube containing EDTA and was stored at −80°C. Genomic DNA was extracted using QiAamp DNA Blood Midi Kit (Qiagen, Germany). The concentration and quality of DNA was quantified by Qubit dsDNA HS Assay Kit (Promega, United States).

### SNP Selection and Genotyping

Genotyping was performed using the Illumina Asian Screening Array (ASA) BeadChip platform covering ∼660k variants across the genome. Imputation was performed with the IMPUTE computer program using the 1000 Genomes Project Han Chinese in Beijing (CHB) population as the reference, with imputation information score >0.90. We intended to replicate 20 single-nucleotide polymorphisms (SNPs) which were identified in the transethnic GWAS by Howles et al. (2019). We also evaluated the association of other 5 SNPs (rs1256328 at ALPL, rs7627468 at CASR, rs12654812 at SLC34A1, rs199565725 and rs219780 at *CLDN* 14) and calcium nephrolithiasis, which were identified in GWAS but not replicated in Chinese Han population (Thorleifsson et al., 2009; Oddsson et al., 2015). A standard quality control procedure was applied to select SNPs for further analysis. SNPs were excluded if they had: 1) genotype call rate <90% or 2) *p* < 0.001 for the Hardy-Weinberg Equilibrium (HWE) test. Therefore, eight SNPs were excluded because of low call rate of genotyping (<90%) and we were able to genotype a total of 17 SNPs. Population stratification analysis was performed by using an ancestry informative marker panel (UT-AIM250) (Wang et al., 2019).

### Statistical Analysis

Quantitative variables were presented as mean ± standard deviation (SD). An independent t test was used to compare the differences between the means of continuous variables. Categorical variables were analyzed using the Chi-square test. Genotype distributions for the SNP were tested for Hardy-Weinberg equilibrium (HWE). The association of SNPs with nephrolithiasis was tested by a Cochran-Armitage trend test. Results are expressed as odds ratio (OR) and 95% confidence intervals (CI). A *p* value lower than 2.94E-03 (0.05/17) was considered statistically significant. SNPs with *p* value less than 0.05 were also considered of interest. Multiple linear regression analyses were used to test association between genotype and clinical parameters, including serum calcium, phosphorus, creatinine, urea, uric acid, etc. with relevant covariates. We conducted association and QTL analyses using the PLINK-1.07 toolset. *p*-values were two tailed. An α of 0.05 was used to claim statistical significance.

## Results

The clinical characteristics of case and control samples were shown in Table 1. Compared with the healthy controls, body mass index (BMI) was significantly higher in patients with calcium nephrolithiasis (*p* = 0.003). There showed no significant difference in the distribution of serum calcium, phosphorus, magnesium, creatinine, and uric acid among the patients and controls.

**TABLE 1:** Baseline characteristics of the study population.

Parameters	Cases (n = 691)	Controls (n = 1008)	*p* Value
Gender (male/female)	467/224	705/303	0.302
Age (years)	50.47 ± 12.55	49.98 ± 12.65	0.431
BMI (kg/m^2^)	24.76 ± 3.30	24.28 ± 3.26	**0.003**
Stone frequency (primary/recurrence)	418/273	-	-
Serum calcium (mmol/L)	2.26 ± 0.12	2.27 ± 0.10	0.062
Serum phosphorus (mmol/L)	1.13 ± 0.21	1.14 ± 0.18	0.293
Serum magnesium (mmol/L)	0.91 ± 0.93	0.89 ± 0.75	0.625
Serum creatinine (μ mol/L)	85.19 ± 41.93	83.64 ± 23.51	0.331
Serum uric acid (mmol/L)	0.360 ± 0.088	0.353 ± 0.077	0.083
Serum albumin (g/L)	42.11 ± 4.11	41.86 ± 4.09	0.217

The bold means p < 0.05.

Population stratification analysis indicated that data of both cases and controls overlapped with Asian populations (Supplementary Figure S1). Therefore, no population stratification was detected between cases and controls. We were able to genotype a total of 17 SNPs (Supplementary Table S1), which were previously reported to be significantly associated with nephrolithiasis in GWAS. Table 2 shows the genotype frequencies of polymorphism among all subjects. The genotype frequencies of 17 SNPs among case and control subjects were distributed in accordance with the Hardy-Weinberg equilibrium (Supplementary Table S2). SNP rs578595 at *WDR*72 was significantly associated with calcium nephrolithiasis in Chinese Han population (*p* < 0.001, OR = 0.617). Four SNPs at 4 loci—— rs12654812 at *SLC34A1* (*p* = 0.0427, OR = 1.170), rs12539707 at *HIBADH* (*p* = 0.0179, OR = 0.734), rs1037271 at *DGKH* (*p* = 0.0096, OR = 0.828) and rs12626330 at *CLDN14* (*p* = 0.0080, OR = 1.213) indicated suggestive associations with calcium nephrolithiasis.

**TABLE 2:** Results of association analysis for calcium nephrolithiasis in Chinese Han population.

Chr	SNP	Gene	Alleles		Cases				Controls				*p* Value	OR	95% CI
			Minor	Major	n (11)^a^	n (12)	n (22)	MAF	n (11)	n (12)	n (22)	MAF			
1	rs10917002	*ALPL*	T	C	60	274	334	0.2949	82	429	487	0.2971	0.8924	0.990	(0.850–1.152)
1	rs1256328	*ALPL*	T	C	33	234	424	0.2171	40	320	647	0.1986	0.1912	1.119	(0.945–1.324)
2	rs780093	*GCKR*	C	T	148	335	208	0.4566	222	507	278	0.4722	0.3703	0.939	(0.818–1.077)
2	rs13003198	*DGKD*	T	C	123	269	214	0.4249	159	433	370	0.3903	0.0546	1.154	(0.997–1.336)
4	rs1481012	*ABCG2*	G	A	79	299	313	0.3307	88	433	471	0.3070	0.1455	1.115	(0.962–1.292)
5	rs12654812	*SLC34A1*	A	G	69	274	348	0.2981	82	373	553	0.2664	**0.0427**	1.170	(1.005–1.362)
6	rs77648599	*SLC22A2*	G	T	1	12	648	0.0106	2	27	904	0.0166	0.1555	0.634	(0.335–1.196)
7	rs12539707	*HIBADH*	T	C	2	97	585	0.0738	3	166	709	0.0980	**0.0179**	0.734	(0.568–0.949)
7	rs12666466	*AQP1*	G	C	7	108	576	0.0883	11	170	827	0.0952	0.4913	0.920	(0.725–1.167)
11	rs4529910	*POU2AF*	G	T	114	293	208	0.4236	184	490	330	0.4273	0.8357	0.985	(0.853–1.137)
13	rs1037271	*DGKH*	T	C	115	302	219	0.4182	210	486	279	0.4646	**0.0096**	0.828	(0.718–0.955)
15	rs578595	*WDR72*	A	C	8	129	438	0.1261	33	310	649	0.1895	<0.001*	0.617	(0.502–0.759)
16	rs889299	*SCNN1B*	A	G	96	290	305	0.3488	134	451	397	0.3661	0.3038	0.927	(0.803–1.071)
17	rs4793434	*SOX9*	G	C	77	312	302	0.3372	130	416	440	0.3428	0.7359	0.975	(0.844–1.128)
19	rs3760702	*GIPC1*	A	G	31	191	392	0.2060	40	329	587	0.2139	0.5970	0.954	(0.800–1.137)
21	rs12626330	*CLDN14*	G	C	155	310	168	0.4897	191	460	302	0.4418	**0.0080**	1.213	(1.052–1.399)
22	rs13054904	*BCR*	A	T	0	49	635	0.0358	2	72	930	0.0379	0.7591	0.944	(0.655–1.362)

n (11), number of subjects with homozygous genotypes for the minor allele; n (12), number of subjects with heterozygous genotypes; n (22), number of subjects with homozygous genotypes for the major allele.

Chr, chromosome; CI, confident interval; MAF, minor allele frequency; OR, odds ratio; SNP, single-nucleotide polymorphism. *p < 0.05/17.

The bold means p < 0.05.

As shown in Table 3, the A allele of rs12654812 was significantly correlated with lower level of serum glucose (*p* = 0.0384). The C allele of rs1037271 was significantly correlated with higher level of serum phosphorus (*p* = 0.0002). The C allele of rs578595 was significantly correlated with higher level of serum creatinine (*p* = 0.0018) and serum glucose (*p* = 0.0085). The G allele of rs12626330 was significantly correlated with higher level of serum uric acid (*p* = 0.0248) and serum carbon dioxide (*p* = 0.0265).

**TABLE 3:** Multiple linear regression analyses for clinical parameters.

	rs12654812			rs12539707			rs1037271			rs578595			rs12626330		
	Beta^1^	s.e.^2^	*P*	Beta	s.e	*P*	Beta	s.e	*P*	Beta	s.e	*P*	Beta	s.e	*P*
eGFR^3^	−0.6906	1.9760	0.7271	−0.4097	3.4150	0.9046	1.7550	1.8140	0.3343	−1.1120	2.9420	0.7058	0.3609	1.7790	0.8394
Serum creatinine	1.1070	2.2720	0.6263	−0.8627	4.1430	0.8351	−0.6331	2.2460	0.7781	11.1500	3.5570	**0.0018**	4.0950	2.1630	0.0588
Serum urea	0.1155	0.1906	0.5447	−0.2427	0.3478	0.4856	−0.1985	0.1889	0.2938	0.2631	0.3056	0.3898	0.0426	0.1125	0.7052
Serum uric acid	0.0049	0.0053	0.3532	0.0078	0.0096	0.4147	0.0042	0.0051	0.4102	−0.0098	0.0080	0.2189	0.0112	0.0050	**0.0248**
Serum sodium	−0.1843	0.1531	0.2291	−0.0265	0.2788	0.9242	0.1501	0.1484	0.3122	−0.0881	0.2395	0.7132	0.1985	0.1456	0.1732
Serum potassium	−0.0737	0.0951	0.4385	−0.0698	0.1735	0.6876	0.1615	0.0948	0.0890	−0.0161	0.1597	0.9200	0.0107	0.0925	0.9083
Serum calcium	−0.0007	0.0072	0.9235	0.0174	0.0131	0.1828	0.0059	0.0069	0.3923	−0.0124	0.0109	0.2558	0.0019	0.0065	0.7761
Serum magnesium	−0.0494	0.0609	0.4176	−0.0585	0.1127	0.6037	0.0193	0.0606	0.7502	−0.0534	0.1014	0.5986	−0.0805	0.0585	0.1693
Serum phosphorus	−0.0039	0.0126	0.7562	0.0035	0.0229	0.8771	0.0460	0.0121	**0.0002**	0.0150	0.0200	0.4532	−0.0065	0.0118	0.5780
Serum chloride	0.3476	0.7096	0.6246	0.4201	1.4030	0.7649	0.2567	0.2140	0.2315	0.5324	0.8509	0.5321	0.3760	0.7242	0.6040
Serum carbon dioxide	−0.2227	0.1703	0.1914	0.4128	0.3098	0.1832	0.1315	0.1652	0.4263	−0.1527	0.2669	0.5675	−0.3602	0.1619	**0.0265**
Serum ALP	−0.1350	1.4900	0.9278	−2.0310	2.7150	0.4548	−2.0600	1.4530	0.1568	−3.1180	2.3540	0.1858	1.0780	1.2440	0.3863
PTH	−3.2670	5.7050	0.5684	9.4190	10.8400	0.3875	−4.7640	5.8240	0.4159	−0.9371	10.5200	0.9293	−2.7360	5.5800	0.6252
Serum 25(OH)D3	3.1600	3.5600	0.3783	3.3860	6.4910	0.6040	−2.7740	3.2960	0.4037	−3.4460	5.2270	0.5127	−3.0890	3.0750	0.3193
BMI	0.0511	0.2019	0.8004	0.0259	0.3666	0.9436	−0.1252	0.1958	0.5229	0.4291	0.3209	0.1817	0.1562	0.1932	0.4191
Serum albumin	−0.1887	0.2502	0.4511	−0.1582	0.4658	0.7342	−0.1432	0.2453	0.5595	−0.2014	0.3700	0.5864	0.1475	0.2393	0.5379
Serum glucose	−0.1579	0.0761	**0.0384**	0.1014	0.1399	0.4689	−0.0890	0.0753	0.2375	0.2855	0.1081	**0.0085**	0.0201	0.0718	0.7799
Serum total cholesterol	−0.0577	0.2219	0.7959	0.6641	0.3903	0.0940	0.1290	0.2273	0.5727	0.3198	0.3467	0.3606	0.0323	0.2581	0.9010
Serum triglycerides	0.0092	0.1142	0.9360	0.1535	0.2057	0.4584	0.0233	0.1119	0.8355	−0.2589	0.1771	0.1498	0.0318	0.1343	0.8139
Serum LDL	−0.4931	0.6252	0.4334	0.2574	1.4050	0.8552	0.3728	0.6377	0.5613	1.6360	0.9911	0.1049	−0.9971	0.7265	0.1759
Serum HDL	−0.0260	0.0415	0.5337	0.0933	0.0909	0.3090	0.0072	0.0404	0.8601	−0.0701	0.0608	0.2551	−0.0303	0.0427	0.4811
Urine calcium	0.0824	0.6251	0.8958	0.4320	1.0930	0.6947	−0.3524	0.6987	0.6170	1.0610	1.4630	0.4739	−0.1075	0.6356	0.8666
Urine phosphorus	1.7620	1.8620	0.3497	−0.9060	3.2940	0.7847	3.6680	2.0420	0.0806	−4.7250	4.8090	0.3337	0.4739	2.0380	0.8174

1 Beta, Regression coefficient.

2 s.e., standard error of mean.

3 eGFR (ml/min/1.73m^2^) = 186 × (serum creatinine/88.41) - 1.154 × age-0.203 (×0.742 if female).

eGFR, estimated glomerular filtration rate; ALP, alkaline phosphatase; PTH, parathyroid hormone; 25(OH)D3, 25-hydroxycholecalciferol; BMI, body mass index; LDL, low-density lipoprotein; HDL, high-density lipoprotein.

The bold means p < 0.05.

## Discussion

In Howles study, which is the largest nephrolithiasis GWAS to date and integrates data from 12,123 stone formers and 417,378 controls from British and Japanese populations, identified 20 loci associated with nephrolithiasis, 7 of which have not previously been reported to associate with nephrolithiasis (Howles et al., 2019). In this study, we evaluated the association of 17 SNPs of 16 loci identified in former GWAS with nephrolithiasis in Chinese Han population. Our results indicated that rs578595 at *WDR*72, rs1037271 at *DGKH*, rs12654812 at *SLC34A1*, rs12539707 at *HIBADH*, and rs12626330 at *CLDN14* were associated with the risk of calcium nephrolithiasis in Chinese Han population. Among them, *WDR72* and *DGKH* were predicted to influence calcium-sensing receptor (CaSR) signaling.

*WDR72* encodes WD repeat containing protein 72 (WDR72), an intracellular protein of 1102 amino acids with no known functional domains except a β-propeller structure composed of WD40 repeat domains in its N-terminus. This domain organization is the characteristic of vesicle coat proteins that mediate membrane deformation complexes to regulate intracellular vesicle trafficking (Katsura et al., 2014). Mutations in *WDR72* have been previously identified as the cause of amelogenesis imperfecta (AI), a hereditary disease that affect tooth enamel formation (El-Sayed et al., 2009). Okada et al. (2012) reported that loss-of-function mutations of *WDR72* result in AI, whereas missense mutations of *WDR72* cause distal renal tubular acidosis (dRTA), possibly without the presence of AI. dRTA is characterized by an impairment of urinary acidification resulting in metabolic acidosis, hypokalemia, and inappropriately elevated urine pH. If not treated, this chronic condition eventually leads to nephrocalcinosis, nephrolithiasis and impaired renal function (D'Ambrosio et al., 2021). Recently, a trans-ethnic GWAS in British and Japanese populations identified that rs578595, an intronic variant in *WDR72*, was significantly associated with nephrolithiasis (Howles et al., 2019). WDR72 are thought to play a role in Clathrin-mediated endocytosis, a process central to sustained intracellular CaSR signaling (Wang et al., 2015). Our results demonstrated that rs578595 was significantly associated with nephrolithiasis in Chinese Han ethnicity. The risk allele of rs578595 was significantly correlated with higher level of serum creatinine, but not correlated with estimated-GFR or level of serum urea. A GWAS in Caucasian population indicated that *WDR72* was associated with renal function and chronic kidney diseases (Kottgen et al., 2010). SNPs on *WDR72* were also found to be associated with the estimated-GFR variance in American Indians (Franceschini et al., 2014). Patients with chronic kidney disease are more likely to develop nephrolithiasis. Moreover, impaired renal function and nephrolithiasis may both probably caused by a dRTA-related mechanism. It is not surprising that rs578595 is correlated with higher level of serum glucose, since *WDR72* has been reported to be associated with higher HbA1c level and poorer blood glucose control (Paterson et al., 2010). Patients with diabetes mellitus are at increased risk for nephrolithiasis, since diabetes might cause stone formation by affecting the composition of urine. It is reported that patients with diabetes excrete more oxalate and have lower urine pH than non-diabetic people (Eisner et al., 2010). Howles et al. (2019) identified rs3760702, ∼300 bp upstream of *GIPC1*, as a significant locus associated with nephrolithiasis. *GIPC1* encodes Regulator of G-protein signaling 19 interacting protein 1 (GIPC1), which is also postulated to play a role in clathrin-mediated endocytosis in CaSR signaling (Shang et al., 2017). In the current study, however, rs3760702 showed insignificant association with nephrolithiasis in Chinese Han population.

*DGKH* encodes for diacylglycerol kinase eta (DGKH), and *DGKD* encodes for diacylglycerol kinase delta (DGKD). DGKD and DGKH phosphorylates diacylglycerol, a component of the intracellular CaSR-signaling pathway inducing CaSR-mediated membrane ruffling and activating protein kinase C (PKC) signaling cascades including mitogen-activated protein kinase (MAPK) and intracellular calcium release (Schlam and Canton, 2017; Gorvin et al., 2018). SNPs of *DGKH* was predicted to promote kidney stone formation by influencing CaSR signaling. In a genome-wide association meta-analysis, *DGKH* was identified to be associated with serum calcium concentrations (O'Seaghdha et al., 2013). In our study, rs1037271, an intronic variant in *DGKH*, showed suggestive association with nephrolithiasis in Chinese Han ethnicity. The risk allele of rs1037271 was significantly correlated with higher level of serum phosphorus. Previously, *DGKD* was identified as a new loci associated with serum calcium in a genome-wide association meta-analysis in Europeans. They characterized the expression of in kidney, and demonstrated that both *DGKD* and *DGKH* were significantly upregulated in response to low calcium diet, which suggested specific involved of these genes in calcium homeostasis (O'Seaghdha et al., 2013). In a GWAS in British and Japanese populations, rs13003198, ∼6 kb upstream of *DGKD*, was identified as a significant locus for calcium nephrolithiasis. Moreover, they verified that *DGKD* knockdown could impair CaSR-signal transduction pathway *in vitro*, and this effect can be rectified with the calcimimetic cinacalcet (Howles et al., 2019). In the current study, SNP rs13003198 was not successfully replicated in Chinese Han population.

*BCR* encodes Breakpoint Cluster Region (BCR) protein, which is a GTPase-activating protein for RAC1 (Rac Family Small GTPase 1). RAC1 activation was postulated to mediate CaSR-induced membrane ruffling (Schlam and Canton, 2017). SNP rs13054904 located ∼110 kb upstream of *BCR*, which was identified to be associated with nephrolithiasis in British population but not Japanese population through GWAS (Howles et al., 2019). We failed to replicate rs13054904 in Chinese Han population, which suggested that this locus predisposed to nephrolithiasis in European rather than East Asian populations.

The *CLDN1*4 gene encodes claudin-14, which belongs to the claudin family of membrane proteins. Claudin-14, a 239-amino acid protein with 4 transmembrane domains and intracellular N and C termini, is an important component of epithelial tight junctions (Tsukita and Furuse, 2000). In the kidney, claudin-14 is predominantly expressed in the thick ascending limb of the Henle’s loop (TALH) where a quarter of filtered calcium is reabsorbed through a passive paracellular pathway involving claudin-14, claudin-16 and claudin-19 (Olinger et al., 2018). CaSR activation is thought to increase expression levels of claudin-14 in the TALH and thereby decrease paracellular calcium reabsorption (Dimke et al., 2013). Moreover, genetic variants may attenuate claudin-14 activity and lead to enhanced paracellular divalent cation reabsorption in the TALH. The first GWAS on nephrolithiasis was reported in 2009, which identified *CLDN14* as a significant locus for nephrolithiasis. Howles et al. (2019) demonstrated that rs12626330, an intronic variant in *CLDN14*, was associated with nephrolithiasis. In this study, rs12626330 showed suggestive association with nephrolithiasis, and rs12626330 was correlated with higher level of serum uric acid and lower level of serum CO_2_. It has been reported that serum CO_2_ level was negatively correlated with the risk of uric acid stone formation (Moreira et al., 2015). Our results suggested that the risk allele of rs12626330 might increase the risk of calcium nephrolithiasis through abnormal metabolism associated with hyperuricemia.

*SLC34A1* gene encodes NPT2a, which is a member of the type II a sodium-phosphate co-transporter family. The NPT2a expressed in the brush border membrane of proximal tubular cells where the bulk of phosphate reabsorption takes place. Mutations in *SLC34A1* have been reported to cause hypophosphatemic nephrolithiasis and osteoporosis in human (Prie et al., 2002). In knockout mice, severe renal phosphate wasting, hypercalciuria and skeletal abnormalities were observed (Beck et al., 1998). In 2012, a GWAS in a Japanese population identified *SLC34A1* as a novel locus associated with nephrolithiasis (Urabe et al., 2012). In 2015, a GWAS in Icelanders reported that common variants of rs12654812 was associated with nephrolithiasis, and rs12654812 associated significantly with decreased serum PTH levels and serum phosphate (Oddsson et al., 2015). In GWAS of Howles et al. (2019) identified rs56235845 as a significant SNP associated with nephrolithiasis in both British and Japanese populations. In our results, we discovered a suggestive correlation between rs12654812, whereas rs56235845 was not significantly associated with nephrolithiasis. The risk allele of rs12654812 was associated with lower level of serum glucose. Presumably, reduction in serum PTH levels associated with rs12654812 may result from a decrease in serum phosphate levels caused by diminished renal reabsorption. Since patients with hyperparathyroidism are usually associated with impair glucose tolerance (Aojula et al., 2021), that kidney stone variance may negatively correlated with serum glucose level through decreased serum PTH.

*HIBADH* encodes 3-hydroxyisobutyrate dehydrogenase (HIBADH). HIBADH is an NAD+ -dependent mitochondrial enzyme that catalyzes oxidation of 3-hydroxyisobutyrate, an intermediate of valine catabolism, to methylmalonate semialdehyde. HIBADH is considered as a key enzyme in the gluconeogenesis pathway (Tasi et al., 2013). *HIBADH* gene was identified as a candidate gene for type 2 diabetes mellitus (Chen et al., 2013). However, its involvement in nephrolithiasis has not been fully elucidated. Howles et al. (2019) first identified rs12539707, an intronic variant in *HIBADH*, as a significant SNP associated with nephrolithiasis in British and Japanese populations. Our results showed suggestive association between rs12539707 and nephrolithiasis in Chinese Han population, which suggests that genes related to glucose metabolism might be involved in the mechanism of nephrolithiasis formation.

To conclude, the results of the present study elucidate that rs578595 at *WDR*72 is significantly associated with calcium nephrolithiasis, whereas rs1037271 at *DGKH*, rs12626330 at *CLDN14*, rs12654812 at *SLC34A1* and rs12539707 at *HIBADH* show suggestive associations with nephrolithiasis in Chinese Han population. As mentioned above, the expression of *CLDN14* localized to the TALH of the kidney was demonstrated to be regulated via the calcium-sensing receptor (CaSR) signaling. Moreover, *WDR72* and *DGKH* are predicted to influence CaSR signaling, but it remains to be confirmed in the kidney. Although further investigation is required, we assumed that the polymorphism of *WDR72*, *DGKH*, and *CLDN14* could increase the risk of calcium nephrolithiasis by influencing the CaSR signaling. Our results emphasized the role of abnormal calcium homeostasis in Chinese patients with calcium nephrolithiasis.

## Data Availability

The original contributions presented in the study are included in the article/Supplementary Material, further inquiries can be directed to the corresponding authors.
