# Supplementary material for: A Genetic Polymorphism in the WDR72 Gene is Associated With Calcium Nephrolithiasis in the Chinese Han Population
Source: Front Genet. 2022 Jul 14;13:897051. doi: 10.3389/fgene.2022.897051 (PMC9333346; doi:10.3389/fgene.2022.897051)
Supplement: Supplementary file 2 [file DataSheet1.DOCX]

***SUPPLEMENTARY MATERIAL***

Catalogue

[1. Supplementary Tables 2](#_Toc10083)

[1.1. Supplemental Table 1. Call rates of genotyped SNPs. 2](#_Toc17599)

[1.2. Supplemental Table 2.](#_Toc29668) *[P](#_Toc29668)*[-values for Hardy-Weinberg equilibrium analysis. 3](#_Toc29668)

[2. Supplementary Figures 5](#_Toc11190)

[2.1 Supplementary Figure 1. Population stratification analysis by using UT-AIM250 panel. 5](#_Toc25515)

1. **Supplementary Tables**

| **CHR** | **SNP** | **N_MISS** | **N_GENO** | **F_MISS** | **Call rate** |
| --- | --- | --- | --- | --- | --- |
| 1 | rs10917002 | 33 | 1699 | 0.0194 | 0.9806 |
| 1 | rs1256328 | 1 | 1699 | 0.0006 | 0.9994 |
| 2 | rs780093 | 1 | 1699 | 0.0006 | 0.9994 |
| 2 | rs13003198 | 131 | 1699 | 0.0771 | 0.9229 |
| 3 | rs7627468 | 187 | 1699 | 0.1101 | 0.8899 |
| 4 | rs1481012 | 16 | 1699 | 0.0094 | 0.9906 |
| 5 | rs12654812 | 0 | 1699 | 0 | 1 |
| 5 | rs56235845 | 224 | 1699 | 0.1318 | 0.8682 |
| 6 | rs1155347 | 775 | 1699 | 0.4562 | 0.5438 |
| 6 | rs77648599 | 105 | 1699 | 0.0618 | 0.9382 |
| 7 | rs12539707 | 137 | 1699 | 0.0806 | 0.9194 |
| 7 | rs12666466 | 0 | 1699 | 0 | 1 |
| 11 | rs4529910 | 80 | 1699 | 0.0471 | 0.9529 |
| 13 | rs1037271 | 88 | 1699 | 0.0518 | 0.9482 |
| 15 | rs578595 | 132 | 1699 | 0.0777 | 0.9223 |
| 16 | rs77924615 | 830 | 1699 | 0.4885 | 0.5115 |
| 16 | rs889299 | 26 | 1699 | 0.0153 | 0.9847 |
| 17 | rs1010269 | 243 | 1699 | 0.1430 | 0.8570 |
| 17 | rs4793434 | 22 | 1699 | 0.0130 | 0.9871 |
| 19 | rs3760702 | 129 | 1699 | 0.0759 | 0.9241 |
| 20 | rs17216707 | 915 | 1699 | 0.5386 | 0.4614 |
| 21 | rs219780 | 1008 | 1699 | 0.5933 | 0.4067 |
| 21 | rs199565725 | 1014 | 1699 | 0.5968 | 0.4032 |
| 21 | rs12626330 | 113 | 1699 | 0.0665 | 0.9335 |
| 22 | rs13054904 | 11 | 1699 | 0.0065 | 0.9935 |

- 1. **Supplemental Table 1. Call rates of genotyped SNPs.**
  2. **Supplemental Table 2. *P*-values for Hardy-Weinberg equilibrium analysis.**

| **CHR** | **SNP** | **TEST** | **A1** | **A2** | **GENO** | **O(HET)** | **E(HET)** | ***P*** |
| --- | --- | --- | --- | --- | --- | --- | --- | --- |
| 1 | rs10917002 | ALL | T | C | 142/703/821 | 0.4220 | 0.4169 | 0.6388 |
| 1 | rs10917002 | AFF | T | C | 60/274/334 | 0.4102 | 0.4159 | 0.7105 |
| 1 | rs10917002 | UNAFF | T | C | 82/429/487 | 0.4299 | 0.4177 | 0.4043 |
| 1 | rs1256328 | ALL | T | C | 73/554/1071 | 0.3263 | 0.3273 | 0.8822 |
| 1 | rs1256328 | AFF | T | C | 33/234/424 | 0.3386 | 0.3399 | 0.9110 |
| 1 | rs1256328 | UNAFF | T | C | 40/320/647 | 0.3178 | 0.3183 | 0.9214 |
| 2 | rs780093 | ALL | C | T | 370/842/486 | 0.4959 | 0.4977 | 0.8838 |
| 2 | rs780093 | AFF | C | T | 148/335/208 | 0.4848 | 0.4962 | 0.5404 |
| 2 | rs780093 | UNAFF | C | T | 222/507/278 | 0.5035 | 0.4985 | 0.8003 |
| 2 | rs13003198 | ALL | T | C | 282/702/584 | 0.4477 | 0.4815 | 0.0055 |
| 2 | rs13003198 | AFF | T | C | 123/269/214 | 0.4439 | 0.4887 | 0.0248 |
| 2 | rs13003198 | UNAFF | T | C | 159/433/370 | 0.4501 | 0.4759 | 0.0908 |
| 4 | rs1481012 | ALL | G | A | 167/732/784 | 0.4349 | 0.4328 | 0.8659 |
| 4 | rs1481012 | AFF | G | A | 79/299/313 | 0.4327 | 0.4427 | 0.5482 |
| 4 | rs1481012 | UNAFF | G | A | 88/433/471 | 0.4365 | 0.4255 | 0.4556 |
| 5 | rs12654812 | ALL | A | G | 151/647/901 | 0.3808 | 0.4026 | 0.0259 |
| 5 | rs12654812 | AFF | A | G | 69/274/348 | 0.3965 | 0.4185 | 0.1728 |
| 5 | rs12654812 | UNAFF | A | G | 82/373/553 | 0.3700 | 0.3908 | 0.0909 |
| 6 | rs77648599 | ALL | G | T | 3/39/1552 | 0.0245 | 0.0278 | 0.0032 |
| 6 | rs77648599 | AFF | G | T | 1/12/648 | 0.0182 | 0.0210 | 0.0672 |
| 6 | rs77648599 | UNAFF | G | T | 2/27/904 | 0.0289 | 0.0327 | 0.0240 |
| 7 | rs12539707 | ALL | T | C | 5/263/1294 | 0.1684 | 0.1595 | 0.0256 |
| 7 | rs12539707 | AFF | T | C | 2/97/585 | 0.1418 | 0.1368 | 0.5710 |
| 7 | rs12539707 | UNAFF | T | C | 3/166/709 | 0.1891 | 0.1767 | 0.0355 |
| 7 | rs12666466 | ALL | G | C | 18/278/1403 | 0.1636 | 0.1677 | 0.3101 |
| 7 | rs12666466 | AFF | G | C | 7/108/576 | 0.1563 | 0.1610 | 0.4733 |
| 7 | rs12666466 | UNAFF | G | C | 11/170/827 | 0.1687 | 0.1723 | 0.4649 |
| 11 | rs4529910 | ALL | G | T | 298/783/538 | 0.4836 | 0.4890 | 0.6843 |
| 11 | rs4529910 | AFF | G | T | 114/293/208 | 0.4764 | 0.4883 | 0.5633 |
| 11 | rs4529910 | UNAFF | G | T | 184/490/330 | 0.4880 | 0.4894 | 0.9486 |
| 13 | rs1037271 | ALL | T | C | 325/788/498 | 0.4891 | 0.4942 | 0.6868 |
| 13 | rs1037271 | AFF | T | C | 115/302/219 | 0.4748 | 0.4866 | 0.5684 |
| 13 | rs1037271 | UNAFF | T | C | 210/486/279 | 0.4985 | 0.4975 | 1 |
| 15 | rs578595 | ALL | A | C | 41/439/1087 | 0.2802 | 0.2772 | 0.7163 |
| 15 | rs578595 | AFF | A | C | 8/129/438 | 0.2243 | 0.2204 | 0.8496 |
| 15 | rs578595 | UNAFF | A | C | 33/310/649 | 0.3125 | 0.3072 | 0.6791 |
| 16 | rs889299 | ALL | A | G | 230/741/702 | 0.4429 | 0.4602 | 0.1237 |
| 16 | rs889299 | AFF | A | G | 96/290/305 | 0.4197 | 0.4543 | 0.0448 |
| 16 | rs889299 | UNAFF | A | G | 134/451/397 | 0.4593 | 0.4641 | 0.7315 |
| 17 | rs4793434 | ALL | G | C | 207/728/742 | 0.4341 | 0.4491 | 0.1741 |
| 17 | rs4793434 | AFF | G | C | 77/312/302 | 0.4515 | 0.4470 | 0.8648 |
| 17 | rs4793434 | UNAFF | G | C | 130/416/440 | 0.4219 | 0.4506 | 0.0478 |
| 19 | rs3760702 | ALL | A | G | 71/520/979 | 0.3312 | 0.3328 | 0.8794 |
| 19 | rs3760702 | AFF | A | G | 31/191/392 | 0.3111 | 0.3272 | 0.2184 |
| 19 | rs3760702 | UNAFF | A | G | 40/329/587 | 0.3441 | 0.3363 | 0.5023 |
| 21 | rs12626330 | ALL | G | C | 346/770/470 | 0.4855 | 0.4969 | 0.3632 |
| 21 | rs12626330 | AFF | G | C | 155/310/168 | 0.4897 | 0.4998 | 0.6333 |
| 21 | rs12626330 | UNAFF | G | C | 191/460/302 | 0.4827 | 0.4932 | 0.5117 |
| 22 | rs13054904 | ALL | A | T | 2/121/1565 | 0.0717 | 0.0713 | 1 |
| 22 | rs13054904 | AFF | A | T | 0/49/635 | 0.0716 | 0.0691 | 1 |
| 22 | rs13054904 | UNAFF | A | T | 2/72/930 | 0.0717 | 0.0728 | 0.6491 |

1. **Supplementary Figures**
   1. **Supplementary Figure 1. Population stratification analysis by using UT-AIM250 panel.**


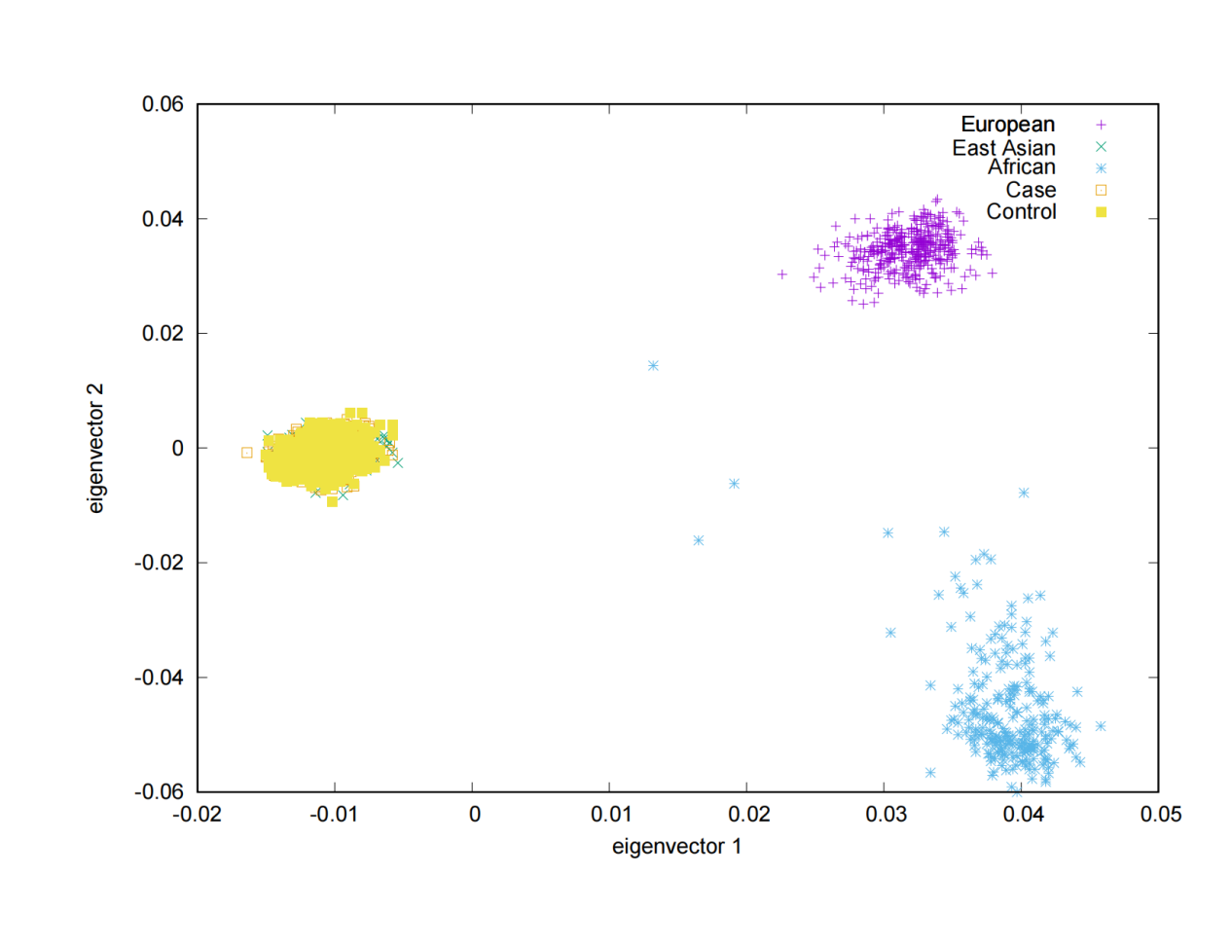


**Supplementary Figure 1 ⎜** Population stratification analysis by using UT-AIM250 panel.
